# Methyl 3-[3-(ethoxy­carbon­yl)thio­ureido]-1*H*-pyrazole-5-carboxyl­ate

**DOI:** 10.1107/S1600536809016742

**Published:** 2009-05-14

**Authors:** Buwen Huang, Pei-Pei Kung, Arnold L. Rheingold, Antonio DiPasquale, Alex Yanovsky

**Affiliations:** a Pfizer Global Research and Development, La Jolla Labs, 10770 Science Center Drive, San Diego, CA 92121, USA; b Department of Chemistry and Biochemistry, University of California, San Diego, 9500 Gilman Drive, La Jolla, CA 92093, USA

## Abstract

The title compound, C_9_H_12_N_4_O_4_S, was proven to be the product of the reaction of methyl 5-amino-1*H*-pyrazole-3-carboxyl­ate with ethyl isothio­cyanato­carbonate. All non-H atoms of the mol­ecule are planar, the mean deviation from the least squares plane being 0.048 Å. The intra­molecular N—H⋯O bond involving the NH-group, which links the thio­urea and pyrazole fragments, closes a six-membered pseudo-heterocyclic ring, and two more hydrogen bonds (N—H⋯O with the participation of the pyrazole NH group and N—H⋯S involving the second thio­urea NH group) link the mol­ecules into infinite chains running along [1
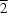
0].

## Related literature

For the structures of similar *N*-pyrazole-substituted thio­urea derivatives, see: Pask *et al.* (2006 ▶); Wen *et al.* (2006 ▶).
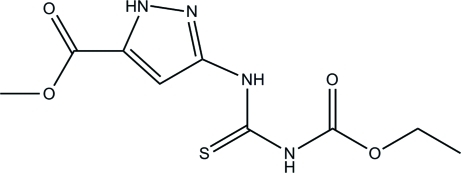


## Experimental

### 

#### Crystal data

C_9_H_12_N_4_O_4_S
*M* *_r_* = 272.29
Triclinic, 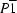

*a* = 8.0855 (8) Å
*b* = 9.0035 (8) Å
*c* = 9.5959 (9) Å
α = 64.510 (1)°
β = 82.294 (1)°
γ = 78.716 (1)°
*V* = 617.39 (10) Å^3^
*Z* = 2
Mo *K*α radiation
μ = 0.28 mm^−1^
*T* = 208 K
0.20 × 0.15 × 0.10 mm

#### Data collection

Siemens P4 diffractometer with APEX CCD detector
Absorption correction: multi-scan (*SADABS*; Bruker, 2001 ▶) *T*_min_ = 0.947, *T*_max_ = 0.973
5852 measured reflections
2653 independent reflections
2255 reflections with *I* > 2σ(*I*)
*R*_int_ = 0.044

#### Refinement

*R*[*F* ^2^ > 2σ(*F* ^2^)] = 0.040
*wR*(*F* ^2^) = 0.113
*S* = 1.04
2653 reflections
166 parameters
H-atom parameters constrained
Δρ_max_ = 0.39 e Å^−3^
Δρ_min_ = −0.28 e Å^−3^

### 

Data collection: *SMART* (Bruker, 1997 ▶); cell refinement: *SAINT* (Bruker, 1997 ▶); data reduction: *SAINT*; program(s) used to solve structure: *SIR2004* (Burla *et al.*, 2005 ▶); program(s) used to refine structure: *SHELXL97* (Sheldrick, 2008 ▶); molecular graphics: *ORTEP-32* (Farrugia, 1997 ▶); software used to prepare material for publication: *WinGX* (Farrugia, 1999 ▶).

## Supplementary Material

Crystal structure: contains datablocks global, I. DOI: 10.1107/S1600536809016742/dn2451sup1.cif

Structure factors: contains datablocks I. DOI: 10.1107/S1600536809016742/dn2451Isup2.hkl

Additional supplementary materials:  crystallographic information; 3D view; checkCIF report

## Figures and Tables

**Table 1:** Hydrogen-bond geometry (Å, °)

*D*—H⋯*A*	*D*—H	H⋯*A*	*D*⋯*A*	*D*—H⋯*A*
N1—H1⋯S1^i^	0.87	2.51	3.347 (1)	161
N2—H2⋯O2	0.87	1.92	2.657 (2)	141
N4—H4⋯O3^ii^	0.87	2.03	2.876 (2)	164

Symmetry codes: (i) 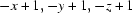; (ii) 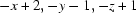.

**Fig. 1.**
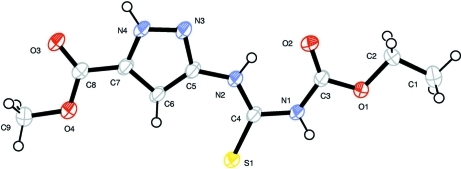
Molecular structure of the title compound showing 50% probability displacement ellipsoids and atom numbering scheme; H atoms are drawn as circles with arbitrary small radius.

**Fig. 2.**
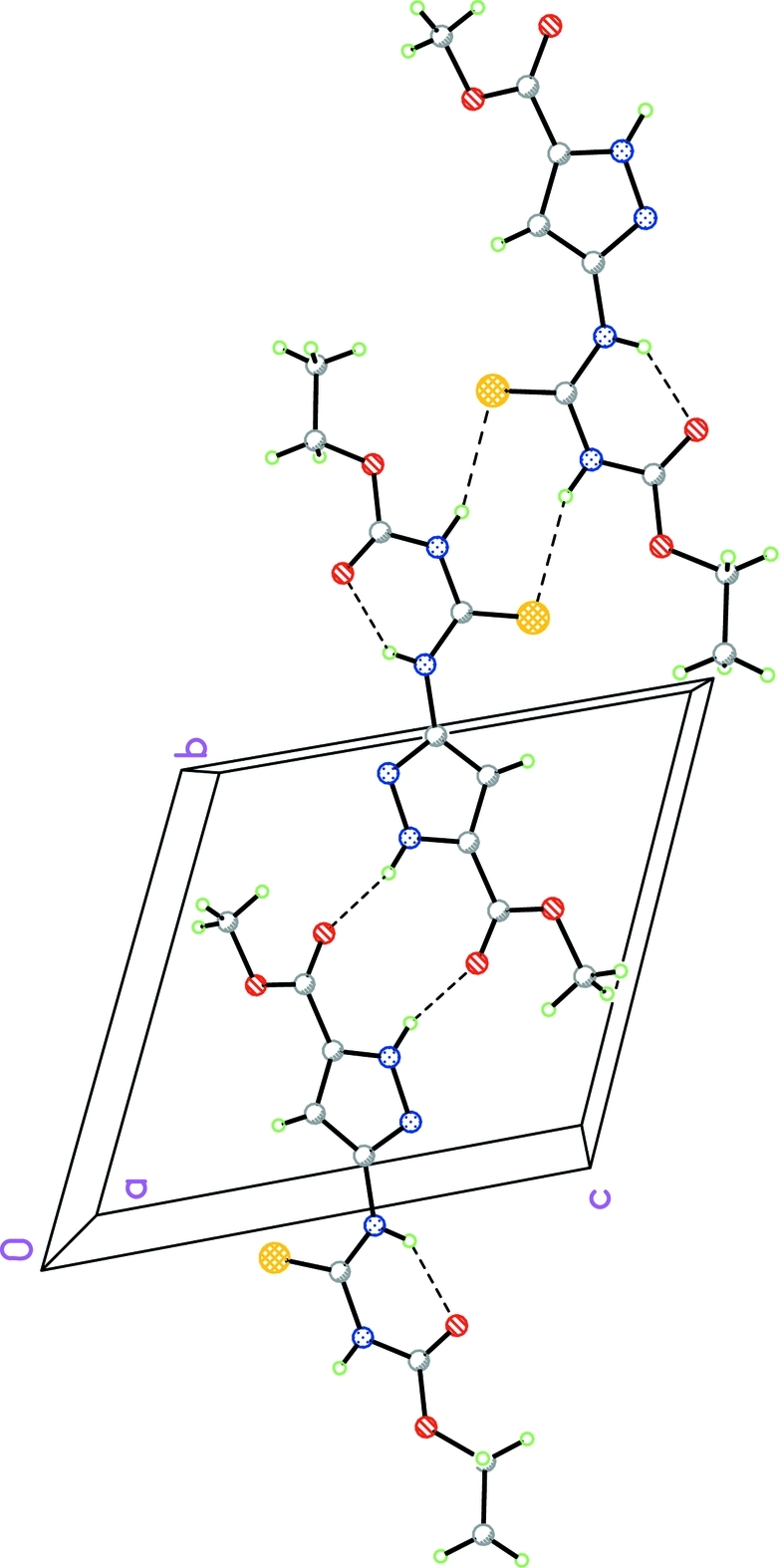
Packing diagram for the title compound viewed approximately along the a axis; H-bonds are shown as dashed lines.

## supplementary crystallographic information

### Comment

The reaction of methyl 5-amino-1*H*-pyrazole-3-carboxylate with ethyl isothiocyanatocarbonate produces the pyrazole-thiourea deivative; its structure was established by the present X-ray study (Fig.1).

All non-H atoms of the molecule are planar (mean deviation from its least squares plane is 0.048 Å), in contrast to previously studied pyrazole-thiourea derivative (Wen *et al.*, 2006), where the pyrazole fragment has a nitrile substituent in position 4 and pyrazole/thiourea fragments form dihedral angle of 46.2°. Another similar compound, where pyrazole has no substituents in position 4 (Pask *et al.*, 2006), is also essentially planar, just like the title compound.

There are three NH-groups in the molecule which are responsible for the formation of three independent H-bonds in the crystal (Table 2). The intramolecular N2—H2···O2 bond closes the 6-membered pseudo-cycle, whereas two intermolecular H-bonds each produce typical centrosymmmetric pairing motive, and their combination thus gives rise to infinite chains running along the [1,-2,0]. direction in the crystal (Fig. 2).

### Experimental

A suspension of methyl 5-amino-1*H*-pyrazole-3-carboxylate (2.0 g, 14.2 mmol) in 10 ml of ethyl acetate and 40 ml of benzene was cooled to 0°C and stirred. To this solution, ethyl isothiocyanatocarbonate (2.04 g, 15.6 mmol) in 10 ml benzene was added dropwise. The resulting reaction mixture was allowed to warm up to room temperature, and stirring was continued for 5 h. The reaction mixture was filtered, and washed with plenty of ether to afford the desired product (3.32 g, 12.2 mmol, 86.0% yield). ^1^H NMR (400 MHz, DMSO-d_6_) δ p.p.m.: 13.99 (br. s., 1 H), 12.12 (br. s., 1 H), 11.48 (br. s., 1 H), 7.51 (s, 1 H), 4.22 (q, J=7.07 Hz, 2 H), 3.85 (s, 3 H), 1.26 (t, J=7.07 Hz, 3 H).

### Refinement

All H atoms were placed in geometrically calculated positions (N—H 0.87 Å, C—H 0.94 Å, 0.97 Å, 0.98 Å, for aromatic, methyl and methylene H atoms respectively) and included in the refinement in riding motion approximation. The *U*_iso_(H) were set to 1.2*U*_eq_ of the carrying atom for aromatic, methylene, methyne and amine groups, and 1.5*U*_eq_ for methyl H atoms.

### Crystal data

**Table:**

C_9_H_12_N_4_O_4_S	*Z* = 2
*M**_r_* = 272.29	*F*(000) = 284
Triclinic, *P*1	*D*_x_ = 1.465 Mg m^−^^3^
Hall symbol: -P 1	Mo *K*α radiation, λ = 0.71073 Å
*a* = 8.0855 (8) Å	Cell parameters from 3767 reflections
*b* = 9.0035 (8) Å	θ = 2.5–27.8°
*c* = 9.5959 (9) Å	µ = 0.28 mm^−^^1^
α = 64.510 (1)°	*T* = 208 K
β = 82.294 (1)°	Block, colorless
γ = 78.716 (1)°	0.20 × 0.15 × 0.10 mm
*V* = 617.39 (10) Å^3^	

### Data collection

**Table:**

Siemens P4 diffractometer with APEX CCD	2653 independent reflections
Radiation source: fine-focus sealed tube	2255 reflections with *I* > 2σ(*I*)
graphite	*R*_int_ = 0.044
φ and ω scans	θ_max_ = 28.2°, θ_min_ = 2.4°
Absorption correction: multi-scan (*SADABS*; Bruker, 2001)	*h* = −5→10
*T*_min_ = 0.947, *T*_max_ = 0.973	*k* = −11→11
5852 measured reflections	*l* = −11→12

### Refinement

**Table:**

Refinement on *F*^2^	Secondary atom site location: difference Fourier map
Least-squares matrix: full	Hydrogen site location: inferred from neighbouring sites
*R*[*F*^2^ > 2σ(*F*^2^)] = 0.040	H-atom parameters constrained
*wR*(*F*^2^) = 0.113	*w* = 1/[σ^2^(*F*_o_^2^) + (0.0521*P*)^2^ + 0.1805*P*] where *P* = (*F*_o_^2^ + 2*F*_c_^2^)/3
*S* = 1.04	(Δ/σ)_max_ = 0.001
2653 reflections	Δρ_max_ = 0.39 e Å^−^^3^
166 parameters	Δρ_min_ = −0.28 e Å^−^^3^
0 restraints	Extinction correction: *SHELXL* (Sheldrick, 2008), Fc^*^=kFc[1+0.001xFc^2^λ^3^/sin(2θ)]^-1/4^
Primary atom site location: structure-invariant direct methods	Extinction coefficient: 0.064 (8)

### Special details

**Table:**

Geometry. All e.s.d.'s (except the e.s.d. in the dihedral angle between two l.s. planes) are estimated using the full covariance matrix. The cell e.s.d.'s are taken into account individually in the estimation of e.s.d.'s in distances, angles and torsion angles; correlations between e.s.d.'s in cell parameters are only used when they are defined by crystal symmetry. An approximate (isotropic) treatment of cell e.s.d.'s is used for estimating e.s.d.'s involving l.s. planes.
Refinement. Refinement of *F*^2^ against ALL reflections. The weighted *R*-factor *wR* and goodness of fit *S* are based on *F*^2^, conventional *R*-factors *R* are based on *F*, with *F* set to zero for negative *F*^2^. The threshold expression of *F*^2^ > σ(*F*^2^) is used only for calculating *R*-factors(gt) *etc*. and is not relevant to the choice of reflections for refinement. *R*-factors based on *F*^2^ are statistically about twice as large as those based on *F*, and *R*- factors based on ALL data will be even larger.

### Fractional atomic coordinates and isotropic or equivalent isotropic displacement parameters (Å^2^)

**Table:**

	*x*	*y*	*z*	*U*_iso_*/*U*_eq_	
C1	0.7861 (3)	0.9454 (2)	−0.0198 (2)	0.0539 (6)	
H1A	0.6653	0.9725	−0.0320	0.081*	
H1B	0.8446	1.0106	−0.1156	0.081*	
H1C	0.8125	0.9706	0.0626	0.081*	
C2	0.8414 (3)	0.7653 (2)	0.0194 (2)	0.0451 (5)	
H2A	0.8135	0.7375	−0.0619	0.054*	
H2B	0.9638	0.7366	0.0301	0.054*	
C3	0.7832 (2)	0.5090 (2)	0.22047 (19)	0.0319 (4)	
C4	0.6784 (2)	0.27556 (19)	0.44802 (18)	0.0285 (4)	
C5	0.7965 (2)	−0.00499 (19)	0.46055 (19)	0.0300 (4)	
C6	0.7316 (2)	−0.1229 (2)	0.5968 (2)	0.0310 (4)	
H6	0.6562	−0.1048	0.6734	0.037*	
C7	0.8050 (2)	−0.2725 (2)	0.59165 (19)	0.0312 (4)	
C8	0.7909 (2)	−0.4462 (2)	0.6958 (2)	0.0328 (4)	
C9	0.6497 (3)	−0.6292 (2)	0.9141 (2)	0.0435 (5)	
H9A	0.6341	−0.6894	0.8550	0.065*	
H9B	0.5509	−0.6267	0.9831	0.065*	
H9C	0.7486	−0.6844	0.9741	0.065*	
N1	0.69023 (19)	0.44206 (16)	0.35749 (16)	0.0322 (3)	
H1	0.6316	0.5127	0.3917	0.039*	
N2	0.77543 (19)	0.16936 (16)	0.39615 (16)	0.0327 (3)	
H2	0.8340	0.2152	0.3099	0.039*	
N3	0.9003 (2)	−0.07384 (17)	0.37579 (17)	0.0360 (4)	
N4	0.9033 (2)	−0.23814 (17)	0.46052 (17)	0.0344 (3)	
H4	0.9626	−0.3141	0.4333	0.041*	
O1	0.75193 (17)	0.67399 (14)	0.16595 (14)	0.0374 (3)	
O2	0.87813 (18)	0.43041 (15)	0.15826 (15)	0.0425 (3)	
O3	0.87755 (18)	−0.56425 (14)	0.67884 (15)	0.0407 (3)	
O4	0.67259 (17)	−0.46002 (15)	0.80911 (15)	0.0397 (3)	
S1	0.54880 (6)	0.22717 (5)	0.60563 (5)	0.03395 (17)	

### Atomic displacement parameters (Å^2^)

**Table:**

	*U*^11^	*U*^22^	*U*^33^	*U*^12^	*U*^13^	*U*^23^
C1	0.0681 (15)	0.0334 (10)	0.0449 (11)	−0.0077 (10)	0.0106 (10)	−0.0060 (8)
C2	0.0512 (12)	0.0338 (9)	0.0352 (9)	−0.0029 (8)	0.0133 (8)	−0.0061 (8)
C3	0.0347 (9)	0.0267 (8)	0.0311 (8)	0.0000 (7)	0.0010 (7)	−0.0119 (6)
C4	0.0312 (9)	0.0247 (7)	0.0300 (8)	0.0008 (6)	−0.0025 (7)	−0.0137 (6)
C5	0.0340 (9)	0.0240 (8)	0.0333 (8)	0.0005 (6)	−0.0013 (7)	−0.0154 (7)
C6	0.0343 (9)	0.0257 (8)	0.0346 (8)	0.0005 (6)	0.0007 (7)	−0.0171 (7)
C7	0.0346 (9)	0.0259 (8)	0.0359 (9)	−0.0010 (7)	−0.0003 (7)	−0.0174 (7)
C8	0.0356 (9)	0.0295 (8)	0.0373 (9)	−0.0036 (7)	0.0003 (7)	−0.0189 (7)
C9	0.0499 (12)	0.0310 (9)	0.0460 (11)	−0.0095 (8)	0.0089 (9)	−0.0147 (8)
N1	0.0393 (8)	0.0234 (7)	0.0306 (7)	−0.0001 (6)	0.0074 (6)	−0.0127 (6)
N2	0.0405 (8)	0.0234 (7)	0.0313 (7)	−0.0012 (6)	0.0061 (6)	−0.0124 (6)
N3	0.0436 (9)	0.0251 (7)	0.0383 (8)	−0.0008 (6)	0.0038 (7)	−0.0160 (6)
N4	0.0403 (9)	0.0256 (7)	0.0396 (8)	−0.0003 (6)	0.0042 (7)	−0.0195 (6)
O1	0.0432 (7)	0.0254 (6)	0.0341 (6)	−0.0013 (5)	0.0113 (5)	−0.0091 (5)
O2	0.0514 (8)	0.0321 (7)	0.0377 (7)	0.0001 (6)	0.0138 (6)	−0.0160 (6)
O3	0.0490 (8)	0.0263 (6)	0.0472 (7)	−0.0015 (6)	0.0073 (6)	−0.0204 (6)
O4	0.0455 (8)	0.0274 (6)	0.0448 (7)	−0.0050 (5)	0.0098 (6)	−0.0174 (5)
S1	0.0400 (3)	0.0257 (2)	0.0328 (2)	−0.00173 (17)	0.00760 (18)	−0.01324 (18)

### Geometric parameters (Å, °)

**Table:**

C1—C2	1.486 (3)	C5—N2	1.401 (2)
C1—H1A	0.9700	C6—C7	1.380 (2)
C1—H1B	0.9700	C6—H6	0.9400
C1—H1C	0.9700	C7—N4	1.343 (2)
C2—O1	1.463 (2)	C7—C8	1.466 (2)
C2—H2A	0.9800	C8—O3	1.214 (2)
C2—H2B	0.9800	C8—O4	1.329 (2)
C3—O2	1.214 (2)	C9—O4	1.452 (2)
C3—O1	1.3278 (19)	C9—H9A	0.9700
C3—N1	1.374 (2)	C9—H9B	0.9700
C4—N2	1.338 (2)	C9—H9C	0.9700
C4—N1	1.387 (2)	N1—H1	0.8700
C4—S1	1.6617 (16)	N2—H2	0.8700
C5—N3	1.340 (2)	N3—N4	1.344 (2)
C5—C6	1.397 (2)	N4—H4	0.8700
			
C2—C1—H1A	109.5	N4—C7—C6	107.60 (14)
C2—C1—H1B	109.5	N4—C7—C8	119.72 (14)
H1A—C1—H1B	109.5	C6—C7—C8	132.67 (16)
C2—C1—H1C	109.5	O3—C8—O4	123.84 (16)
H1A—C1—H1C	109.5	O3—C8—C7	123.32 (16)
H1B—C1—H1C	109.5	O4—C8—C7	112.84 (14)
O1—C2—C1	106.83 (15)	O4—C9—H9A	109.5
O1—C2—H2A	110.4	O4—C9—H9B	109.5
C1—C2—H2A	110.4	H9A—C9—H9B	109.5
O1—C2—H2B	110.4	O4—C9—H9C	109.5
C1—C2—H2B	110.4	H9A—C9—H9C	109.5
H2A—C2—H2B	108.6	H9B—C9—H9C	109.5
O2—C3—O1	125.25 (16)	C3—N1—C4	127.95 (13)
O2—C3—N1	125.62 (15)	C3—N1—H1	116.0
O1—C3—N1	109.13 (13)	C4—N1—H1	116.0
N2—C4—N1	114.69 (14)	C4—N2—C5	129.41 (14)
N2—C4—S1	126.73 (12)	C4—N2—H2	115.3
N1—C4—S1	118.59 (11)	C5—N2—H2	115.3
N3—C5—C6	112.89 (14)	C5—N3—N4	103.49 (14)
N3—C5—N2	114.22 (15)	C7—N4—N3	112.76 (13)
C6—C5—N2	132.89 (15)	C7—N4—H4	123.6
C7—C6—C5	103.26 (14)	N3—N4—H4	123.6
C7—C6—H6	128.4	C3—O1—C2	116.14 (14)
C5—C6—H6	128.4	C8—O4—C9	115.46 (13)

### Hydrogen-bond geometry (Å, °)

**Table:**

*D*—H···*A*	*D*—H	H···*A*	*D*···*A*	*D*—H···*A*
N1—H1···S1^i^	0.87	2.51	3.347 (1)	161
N2—H2···O2	0.87	1.92	2.657 (2)	141
N4—H4···O3^ii^	0.87	2.03	2.876 (2)	164

Symmetry codes: (i) −*x*+1, −*y*+1, −*z*+1; (ii) −*x*+2, −*y*−1, −*z*+1.

## References

[bb1] Bruker (1997). *SMART* and *SAINT* Bruker AXS Inc., Madison, Wisconsin, USA.

[bb2] Bruker (2001). *SADABS* Bruker AXS Inc., Madison, Wisconsin, USA.

[bb3] Burla, M. C., Caliandro, R., Camalli, M., Carrozzini, B., Cascarano, G. L., De Caro, L., Giacovazzo, C., Polidori, G. & Spagna, R. (2005). *J. Appl. Cryst.* **38**, 381–388.

[bb4] Farrugia, L. J. (1997). *J. Appl. Cryst.* **30**, 565.

[bb5] Farrugia, L. J. (1999). *J. Appl. Cryst.* **32**, 837–838.

[bb6] Pask, C. M., Camm, K. D., Kilner, C. A. & Halcrow, M. A. (2006). *Tetrahedron Lett.* 2531–2534.

[bb7] Sheldrick, G. M. (2008). *Acta Cryst.* A**64**, 112–122. 10.1107/S0108767307043930 18156677

[bb8] Wen, L.-R., Li, M., Zhou, J.-X. & Liu, P. (2006). *Acta Cryst.* E**62**, o940–o941.

